# Meta-analysis of gene expression and integrin-associated signaling pathways in papillary renal cell carcinoma subtypes

**DOI:** 10.18632/oncotarget.12390

**Published:** 2016-10-01

**Authors:** Kai Zhang, Hang-Mao Lee, Gong-Hong Wei, Aki Manninen

**Affiliations:** ^1^ Biocenter Oulu, Oulu Center for Cell-Matrix Research, Faculty of Biochemistry and Molecular Medicine, University of Oulu, Oulu 90220, Finland

**Keywords:** meta-analysis, papillary renal cell carcinoma, gene expression, integrin, gender

## Abstract

Papillary renal cell carcinoma (PRCC) is the second most common renal cell carcinoma (RCC) that can be further subdivided into type 1 (PRCC1) and type 2 (PRCC2) RCCs based on histological and genetic features. PRCC2 is often more aggressive than PRCC1. While integrin-associated protein complexes mediate tumorigenesis and metastases in many types of cancers it is not known whether integrin-mediated signaling impacts PRCC and differs between PRCC1 and PRCC2. In this study, we combined the analysis of five PRCC gene expression datasets derived from Gene Expression Omnibus (GEO) and The Cancer Genome Atlas (TCGA) by using integrative bioinformatics pipelines. We found 1475 differentially expressed genes among which 37 genes were associated with integrin pathways. In comparison with PRCC1, PRCC2 cases showed upregulated expression of α5-integrin (*ITGA5*) whereas the expression of α6- (*ITGA6*) and β8-integrins (*ITGB8*) was downregulated. Because PRCC2 occurs more frequently in men, the meta-analysis was extended to explore the gender effects. This analysis revealed 8 genes but none of them was related to integrin pathways suggesting that other mechanisms than integrin-mediated signaling underlie the observed gender differences in the pathogenicity of PRCC2.

## INTRODUCTION

The second most common histological subtype of renal cell carcinomas (RCC) is the papillary RCC that accounts for 10–20 percent of all renal cancer cases [1]. Papillary renal cell carcinoma (PRCC) has two subtypes defined by different histological features. PRCC1 shows both papillae and tubular structures covered by small cells with scanty cytoplasm and small oval nuclei. PRCC2 indicates only papillary structures covered by large cells with abundant eosinophilic cytoplasm and large, spherical nuclei with prominent nucleoli [2]. PRCC2 is often a more aggressive disease that is associated with less differentiated histology phenotype, high number of nodal and distant metastases and worse survival rates comparing with PRCC1 [3–4]. PRCC1 and PRCC2 have distinct genetic backgrounds [5]. While overexpression or activating mutations of MET proto-oncogene encoding for a hepatocyte growth factor receptor (HGFR) are common in PRCC1, PRCC2 has been associated with activation of the NRF2-ARE pathway and CDKN2A silencing [6–8]. Interestingly, differential expression of components regulating cell-extracellular matrix (ECM) interactions has also been implicated in PRCC1 [9]. However, how cell-ECM interactions are modulated in PRCC2 remains unknown.

Integrins are heterodimeric cell surface receptors that mediate interactions between cells and the ECM [10]. In solid tumors integrins regulate cancer initiation, stemness, drug resistance and metastasis by regulating the assembly of large multiprotein complexes [11]. These integrin-mediated complexes not only facilitate cells to adhere to the ECM but also convey various signals between cells and their immediate microenvironment to regulate cell behavior [10–11]. Jones *et al.* reported that abnormal chemokine receptor signaling modulates the activity of α3-, α5-, β1-, β3- and β4-integrins thereby regulating the adhesive properties of clear cell renal cell carcinoma (ccRCC) cells *in vitro* [12]. However, possible contributions of modified integrin pathways underlying the histological differences between PRCC1 and PRCC2 have not been thoroughly addressed. Intriguingly, PRCC2 occurs more frequently and is more aggressive in male patients when compared with female patients, but the mechanism remains largely unknown [13–15]. In order to address these issues, we combined available RNA-seq and microarray datasets of PRCC samples from GEO and TCGA and compared the mRNA expression profiles of PRCC1 and PRCC2. To get a comprehensive insight into molecular mechanisms and differences between PRCC1 and PRCC2, we performed a meta-analysis of gene expression focusing on the integrin-associated pathways using these large GEO and TCGA datasets. Our analysis highlighted differential regulation of integrin pathways between PRCC1 and PRCC2 but found no significant gender-associated changes in integrin pathway genes between male and female patients with PRCC2.

## RESULTS

### Meta-analysis of differentially expressed genes between PRCC1 and PRCC2

**Figure 1:**
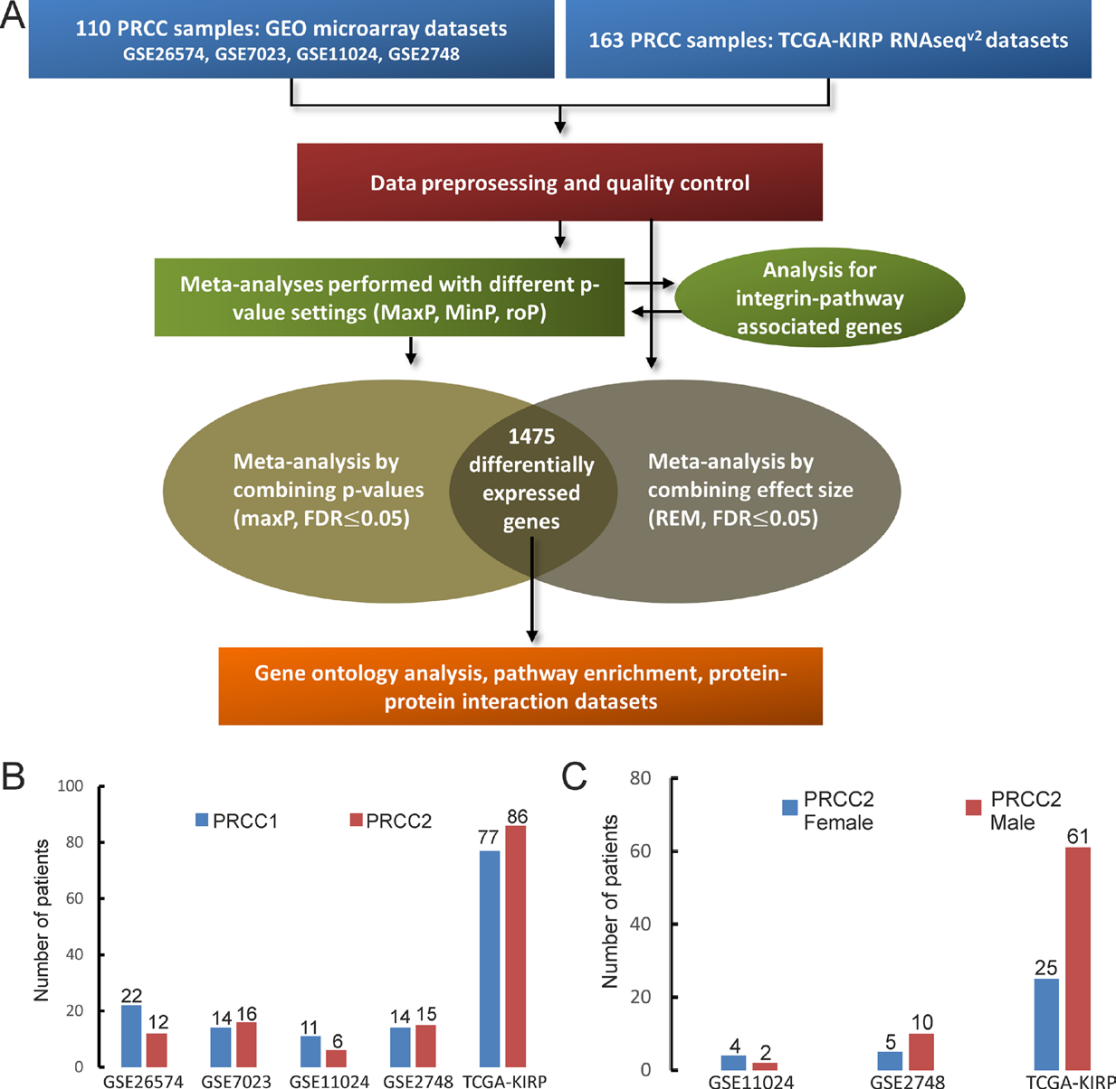
Overview of the meta-analysis and the PRCC datasets (**A**) A scheme of the meta-analysis workflow. (**B**) The number of histologically classified PRCC1 and PRCC2 samples in the five datasets. (**C**) The number of female (blue) and male (red) PRCC2 patients in the three datasets where this information was available.

**Figure 2:**
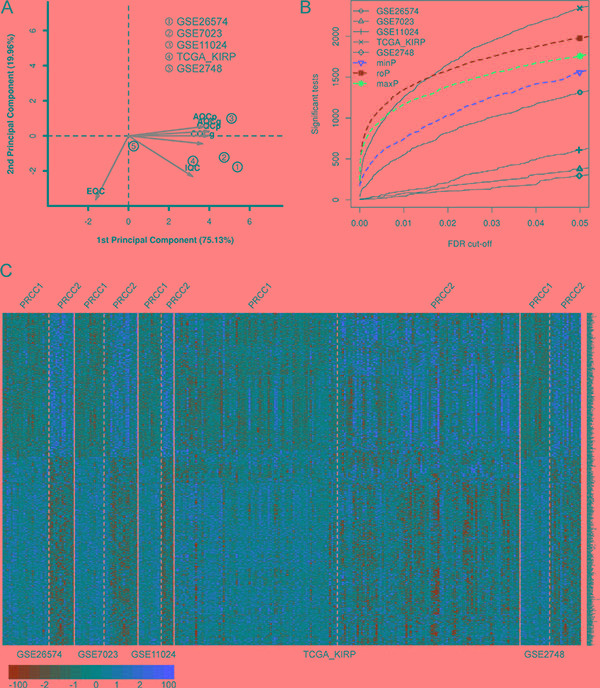
Meta-analysis of differentially expressed genes between PRCC1 and PRCC2 by combining *p*-values (**A**) PCA biplot of quality control measures in five PRCC studies. (**B**) The number of differentially expressed genes plotted as a function of false discovery rate FDR in the analysis of five different datasets and the three different meta-analysis algorithms (maxP, minP and roP). (**C**) A heat map representation of DE genes in PRCC1 and PRCC2 samples subjected to maxP meta-analysis (FDR = 0.05).

**Figure 3:**
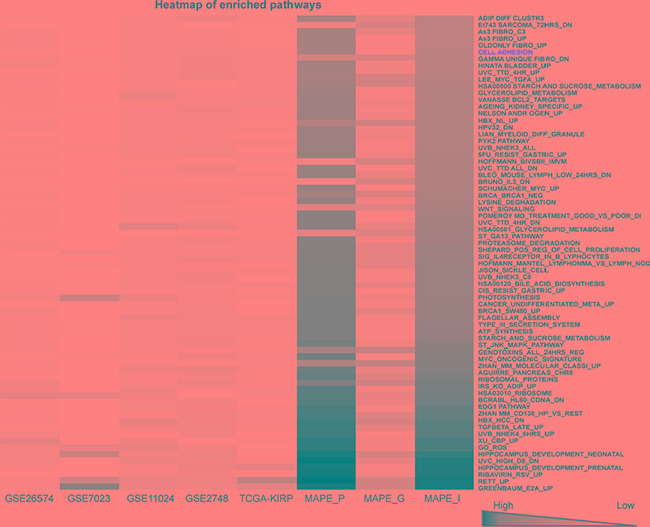
A heatmap of enriched pathways from maxP meta-analysis by combining *p*-values A heatmap showing 115 enriched pathways detected by MAPE_I maxP under *q*-value = 0.2 threshold with the 1758 DE genes between PRCC1 and PRCC2 cases. Cell adhesion pathway is highlighted in red color.

**Figure 4:**
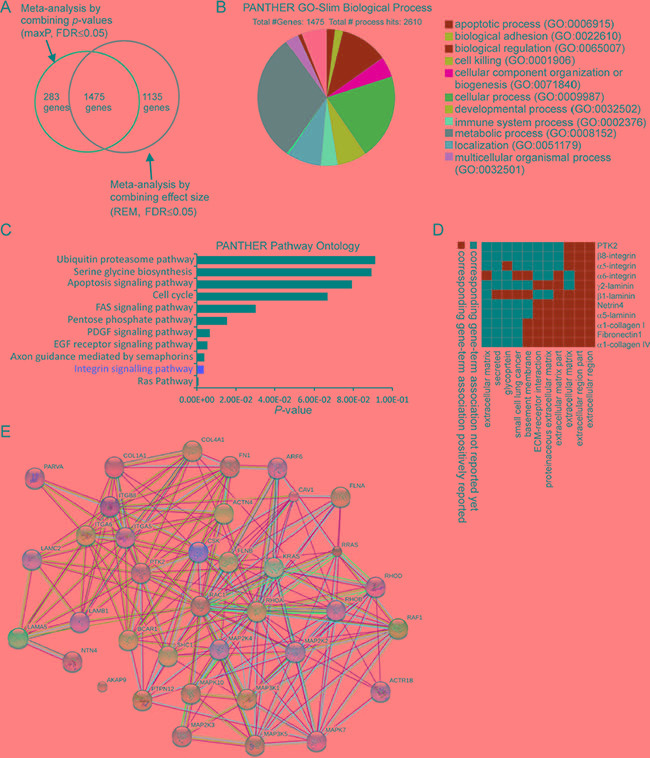
Comparative meta-analysis of differentially expressed genes between PRCC1 and PRCC2 samples (**A**) A Venn Diagram showing the overlap of DE genes detected by two meta-analyses, one combining the *p*-values (maxP) and another combining the effect sizes (REM). (**B**) PANTHER GO-Slim biological process analysis revealing 11 major functional categories associated with the 1475 DE genes. Biological adhesion is one of the major categories. (**C**) PANTHER pathway ontology analysis highlighting integrin pathway as the second-most enriched pathway. (**D**) A functional annotation clustering 2D report highlighting the cell adhesion related pathways associated with the integrin-related DE genes from the meta-analysis. (**E**) Protein-protein functional interaction network including 37 integrin pathway-associated genes extracted from the meta-analysis.

Four microarray datasets of PRCC patient samples for which matched clinical information was available were obtained from GEO by using GEOquery [16]. In addition, one RNA-seq dataset of PRCC samples was obtained from TCGA-kidney renal papillary cell carcinoma (KIRP) database by using TCGA-assembler [17] (Figure 1A and Table 1). After removing non-PRCC1/PRCC2 (or unidentified) samples and PRCC cases with mixed subtyping, a total of 138 PRCC1 and 135 PRCC2 samples were selected for further analysis (Figure 1A–1C). PCA biplots of the six quality control criteria and the five PRCC datasets supported the inclusion of all of the datasets for meta-analysis (Figure 2A and Table 2). Three main meta-analysis methods by combining *p*-value in MetaDE package were employed: 1) Maximum *p*-value (maxP), 2) Minimum *p*-value (minP) and 3) r-th ordered *p*-value (roP) (Figure 2B) [18]. 1758, 1558 and 1976 differentially expressed (DE) genes were detected by maxP, minP and roP evaluation criteria, respectively, using detection competency curves and false discovery rate (FDR) cut-off less than 0.05 (Figure 2B, 2C and Table 3). All of the three analyses highlighted integrin pathways with significant overlap such that 37% of the DE genes within integrin pathways were shared by all three analyses and 55% were shared between two out of three analyses (Supplementary Figure S1A). Meta-analysis performed with maxP criteria, however, identified more integrin pathway-related DE genes than the other two and was thus selected for further analysis. All the 1758 DE genes obtained with maxP criteria were analyzed by using meta-analysis for pathway enrichment (MAPE) within the MetaPath package to reveal cellular pathways differentially regulated between PRCC1 and PRCC2 [19]. MAPE analysis identified 115 enriched pathways when the MAPE_I method (threshold set to 0.2) which integrates gene and pathway information from the DE genes was employed (Figure 3) [19]. Cell adhesion was one of the pathways appearing from this analysis but not among the most prominently enriched pathways. To further refine the list of DE genes between the PRCC subtypes we employed a random-effect model (REM) meta-analysis approach that estimates the differences in gene expression across all the different datasets by combining the individual effects sizes into a meta-effect size (ES) [18–20]. To this end MetaDE.ES was applied for the raw data in the five datasets (Table 1) resulting in 2610 DE genes when the FDR threshold was set to 0.05 (Supplementary Figure S1B and Table 3). Finally, the DE genes obtained from each of these two meta-analyses were plotted into a Venn diagram that revealed 1475 DE genes common to both maxP meta-analysis method that combines *p*-values and REM meta-analysis method that combines effect sizes (Figure 4A).

**Table 1:** The source data for each of the datasets used in this study

Source	Platform	GEO Accession
Ooi A et al 2011	GPL11433	GSE26574
Furge KA et al 2011	GPL4866	GSE7023
Kort EJ et al 2008	GPL6671	GSE11024
Yang XJ et al 2013	GPL570	GSE2748
TCGA-KIRP 2016	IlluminaHiseq RNASeqV2	NA

GEO = Gene Expression Omnibus. KIRP= Kidney renal papillary cell carcinoma.

**Table 2:** The quality control analysis for PRCC1 and PRCC2 in the different datasets

Number	Study	IQC	EQC	CQCg	CQCp	AQCg	AQCp	Rank
1	GSE26574	7.39	3.7	307.65	307.65	307.65	188.44	2
2	GSE2748	4.91	3.82	307.65	307.65	270.54	161.59	2.42
3	GSE7023	2.85	0.06*	307.65	307.65	307.65	182.73	2.83
4	GSE11024	3.79	3.82	307.65	224.15	175.48	68.07	3.25
5	TCGA_KIRP	0.61*	3.82	33.09	70.3	19.52	31.94	4.5

* *p*-value not significant after Bonferroni correction.

**Table 3:** The number of differentially expressed genes in the datasets

Cut-off	GSE26574	GSE7023	GSE11024	TCGA_KIRP	GSE2748	minP	roP	maxP REM
*p* = 0.01	1371	642	891	2021	648	1389	1758	1587 2225
*p* = 0.05	2388	1402	1794	3126	1469	2322	2438	2162 2941
FDR = 0.01	546	78	106	1250	44	744	1347	1168 1837
FDR = 0.05	1314	377	609	2343	294	1558	1976	1758 2610

minimum *P*-value (minP), maximum *P*-value (maxP), rth ordered *P*-value (roP).

### Differential regulation of the integrin pathway signaling between PRCC1 and PRCC2

The 1475 DE genes obtained from the overlay of *p*-value and ES combined meta-analysis were further examined using PANTHER gene ontology-slim biological process analysis. This analysis revealed that metabolic process and cellular process were among the top enriched categories (Figure 4B). PANTHER pathway ontology of the 1475 DE genes demonstrated 37 genes related to integrin pathway which was the second most enriched pathway (Figure 4C). These results were corroborated by similarly overlapping the DE genes from each of the two remaining meta-analyses based on *p*-values (minP and roP) with the DE genes obtained from REM meta-analysis. As expected, PANTHER pathway ontology analysis found integrin signaling pathway to be enriched within shared DE genes between REM and minP (1265 genes) and REM and roP (1300 genes) meta-analyses (Supplementary Figure S1C and S1E).

**Figure 5:**
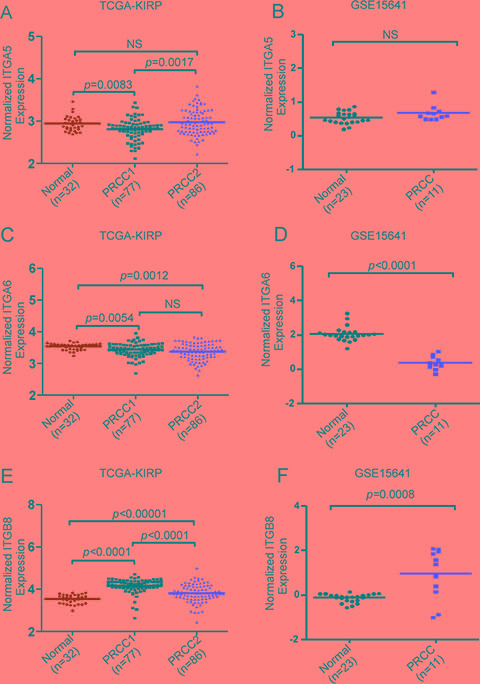
Analysis of the expression levels of ITGA5, ITGA6 and ITGB8 in healthy controls and in PRCC1 and PRCC2 patient samples (**A**) Relative expression levels of ITGA5 in the kidney from healthy control, PRCC1 and PRCC2 in the TCGA-KIRP dataset and (**B**) in healthy controls and PRCC patients from GSE15641 dataset where matched clinical information was not available [26]. (**C**) Relative expression levels of *ITGA6* in the TCGA-KIRP and (**D**) the GSE15641 datasets. (**E**) Relative expression levels of *ITGB8* in the TCGA-KIRP and (**F**) the GSE15641 datasets. Mann-Whitney U tests were performed to assess statistical significance of the observed differences between different sample groups in A–F. The horizontal lines represent median values.

A functional annotation clustering 2D view report of the integrin pathway associated genes from the maxP/REM overlapping dataset was visualized by using DAVID functional annotation tool (Figure 4D). These genes included α5 (*ITGA5*)- and β8 (*ITGB8*)-integrins, both of which are RGD-binding receptors associated with epithelial-to-mesenchymal transition (EMT) [21–22][23]. Moreover, α6-integrin (*ITGA6*), a laminin receptor that regulates epithelial cell polarity and growth was found to be differentially regulated between PRCC1 and PRCC2 (Table 4) [24–25]. Finally, STRING interaction network analysis was performed with these 37 integrin pathway-related genes. This analysis revealed highly connected functional protein-protein interaction network that included all but one of the 37 DE genes (Figure 4E). To relate the differential expression of the three integrin genes in PRCC patients to healthy controls we extracted the expression data from two studies, the TCGA-KIRP study based on RNA-seq data which was also included in our meta-analysis and an independent study by Jones *et al.* (GSE15641) [7, 26]. This analysis revealed that *ITGA5* was significantly downregulated in PRCC1 patients when compared with healthy controls (Figure 5A). In contrast, a tendency for modestly elevated levels of *ITGA5* expression was noted for PRCC2 although this was not statistically significant (Figure 5A). *ITGA5* expression levels in unclassified PRCC patients in the GSE15641 dataset tended to be higher than in healthy controls but this difference was not statistically significant (Figure 5B). Both studies were consistent with significant downregulation of *ITGA6* expression that was particularly evident in PRCC2 patients (Figure 5C). The GSE15641 dataset also displayed a robust downregulation of *ITGA6* in the PRCC samples (Figure 5D). In contrast to reduced *ITGA6* levels, upregulation of *ITGB8* expression was observed in PRCC patients with the highest expression levels seen in PRCC1 patients (Figure 5E). The GSE15641 dataset similarly showed significant upregulation of *ITGB8* in PRCC patient samples (Figure 5F). Taken together, the combined meta-analysis extracted three candidate integrin genes whose differential regulation may underlie the different pathogenic properties of the two PRCC subtypes. Comparing with TCGA-KIRP study, the expression pattern of these three integrins predicts that the majority of the cancer samples in the GSE15641 dataset, where matched clinical information was not available, belong to the PRCC subtype 2.

**Table 4:** Differentially expressed genes of the integrin pathway in PRCC1 and PRCC2

PRCC2 > PRCC1	*PTK2 SHC1 ACTN4 CAV1 COL1A1 COL4A1 FLNA ITGA5 MAP2K2 PARVA RHOD RRAS*
PRCC2 < PRCC1	*PTPN12 RHOA RHOB RAC1 ARF6 AKAP9 ACTR1B CSK BCAR1 FLNB KRAS RAF1 FN1 ITGA6 ITGB8 MAP2K3 MAP2K4 MAP3K1 MAP3K5 NTN4 LAMA5 LAMB1 LAMC2 MAPK10 MAPK7*

PRCC1 = Papillary renal cell carcinoma type1, PRCC2 = Papillary renal cell carcinoma type2.

### Analysis of the gender specific differentially expressed genes in PRCC2 patients

**Figure 6:**
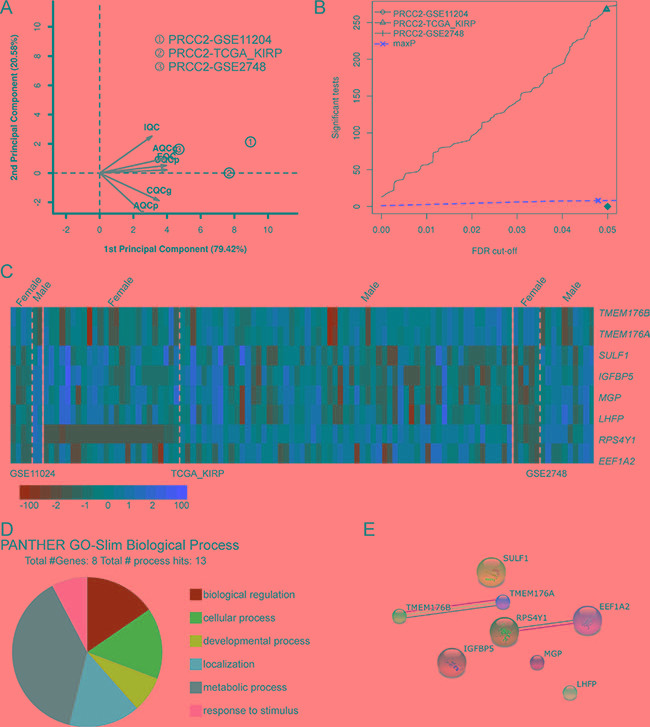
Comparative meta-analysis of DE genes in female and male PRCC2 patients (**A**) PCA biplot of quality control measures in the three PRCC studies after removing non-PRCC2 samples. (**B**) The detection competency curves of DE genes plotted against false-discovery rate in analyses of the three individual datasets and the maxP meta-analyses approaches. (**C**) A heat map representation of DE genes (FDR = 0.05) between female and male PRCC2 patients. (**D**) Molecular function analysis of the 8 DE genes revealed by the meta-analysis. (**E**) Protein-protein interaction network analysis of the 8 DE genes in female and male PRCC2 patients.

Clinical data shows that PRCC2 is more frequent and tends to be more aggressive in male patients [27]. To address the possible underlying genetic factors we subjected male and female PRCC2 patient data for meta-analysis to study the DE genes. Given that information on gender was not available for two GEO datasets, the remaining three datasets (GSE11024, GSE2748 and TCGA-KIRP) were included for further analysis (Figure 1C). After removing PRCC1 cases the selected datasets consisted of 34 female and 73 male PRCC2 samples. To provide objective quality control and inclusion/exclusion criteria of the filtered datasets for meta-analysis, we performed MetaQC and runQC packages in R. All of the three datasets passed the quality control test and were included for further analysis (Figure 6A and Table 5). maxP meta-analysis detected 8 genes that were differentially regulated between female and male PRCC2 samples using a FDR cut-off under 0.05 (Figure 6B). The expression patterns of the 8 genes in the patient samples were visualized in a heat map (Figure 6C). The PANTHER GO-Slim biological process analysis revealed that metabolic process was the major functional category associated with the 8 genes (Figure 6D). STRING Interaction Network analysis of the extracted genes showed that *RPS4Y1* interacted with translation elongation factor *EEF1A2* (Figure 6E). However, *RPS4Y1* is a Y-chromosome-specific gene and when a similar meta-analysis addressing the gender-dependent differences in gene expression was performed for PRCC1 patients where no gender bias has been reported, *RPS4Y1* was also detected among the DE genes (Supplementary Figure 2). Therefore, it is unlikely that elevated *RPS4Y1* expression is associated with the more aggressive pathogenesis of PRCC2 in male patients. In addition to *RSP4Y1*, the analysis of gender-specific DE genes in PRCC2 patients also highlighted interaction of two members of the membrane-spanning 4-domain family, subfamily A (*MS4A*) transmembrane (*TMEM*)-176A and 176B that have been found to be deregulated in multiple cancer types [28]. However, none of the genes were associated with cell adhesion or integrin pathways.

**Table 5:** Quality Control Result for PRCC2 based on gender difference

Number	Study	IQC	EQC	CQCg	CQCp	AQCg	AQCp	Rank
1	GSE2748g	20	3.52	7.83	24.43	5.16	4.59	1.5
2	GSE11024g	4	3.3	9.84	16.25	2.56	8.9	1.83
3	TCGA_KIRPg	2.67	3.7	0.11*	0.88*	0.16*	0.26*	2.67

* *p*-value not significant after Bonferroni correction.

## DISCUSSION

High-throughput genomic datasets of PRCC patient material have accumulated in the public databases such as GEO and TCGA. Meta-analysis combining these datasets helps to increase the statistical power in mining and explaining the underlying mechanisms driving pathogenesis of PRCC subtypes. Here a meta-analysis was performed to extract the differentially expressed genes between PRCC1 and PRCC2 with a particular focus on the integrin-related pathways. The combined meta-analysis extracted 1475 DE genes, 37 of which were associated with integrin pathway. The more aggressive PRCC2 was associated with modest upregulation of *ITGA5*, a fibronectin receptor known to promote tumor progression in ccRCC [29]. Cell biological data supports a positive role for α5β1-integrin in promoting mesenchymal cell migration and cancer cell invasion [30–31]. Ligation of α5β1-integrins have also been reported to support cell survival in suboptimal growth conditions [32–33]. It is likely that upregulated *ITGA5* expression in part contributes to the metastatic properties of PRCC2. *ITGA6* expression was significantly downregulated in PRCC samples, especially in PRCC2, when compared with normal kidneys (Figure 5C and 5D). α6-integrin is a laminin receptor which delineates the basal membrane of polarized epithelial cells and synergizes with growth signals to support cell proliferation and migration [34]. Twist-mediated transformation of renal cancer cells led to reduced expression of *ITGA6* [35]. Reduced expression of α6β4-integrin may enhance cancer cell dissemination from primary tumors and has been reported to positively correlate with prostate cancer progression [36]. However, increased expression of *ITGA6* was reported for metastatic cells of many other solid cancer types [37][38]. Interestingly, α6-integrin is highly expressed in stem cells including cancer stem cells [39]. Our finding on decreased *ITGA6* levels raise the possibility that enhanced dissemination of kidney cancer cells could in part explain the aggressive pathogenesis of PRCC2. However, further functional studies are required to confirm these findings. *ITGB8* was the most robustly upregulated integrin in both PRCC datasets. Especially high levels of *ITGB8* were seen in PRCC1 samples. *ITGB8* forms an αVβ8-integrin heterodimer that plays a critical role in activation of latent TGF-β [40]. TGFβ-activation in turn drives epithelial-to-mesenchymal transition which may directly contribute to cancer cell migration and growth. TGFβ can function as both tumor suppressor and oncogene depending on other coinciding signals [41]. How elevated TGFβ-signaling might regulate PRCC pathogenesis is not known but it has been reported that TGFβ activation modulates metastatic properties of some RCCs [42–43]. TGFβ activation appears to suppress growth of ccRCC tumors but the effect on PRCC remains poorly understood [44].

A meta-analysis was performed to identify gender-specific genes which may explain the different incidence and aggressiveness of PRCC2 between male and female patients. *RPS4Y1*, *EEF1A2*, *TMEM176A* and *TMEM176B* were preferentially up-regulated in male patients. *RPS4Y1*, a Y chromosome-linked gene [45], was found to functionally interact with a translation factor *EEF1A2*. While these proteins were linked to metabolic processes *EEF1A2* may indirectly associate with cell adhesion related pathways as it has been shown to regulate MMP-9 expression and thereby influence migration and metastatic properties of pancreatic cancer cells [46]. Our meta-analysis approach revealed three key integrin subunits that were differentially associated with PRCC subtypes. An important limitation of the current study is that integrin activation and stability is frequently regulated at post-translational level. Therefore inclusion of proteomic data as well as functional cell biological studies are required for future studies to confirm and validate the specific roles of α5-, α6- and β8-integrins highlighted here by genetic meta-analysis.

## MATERIALS AND METHODS

### Data description

173 datasets were found in GEO series (http://www.ncbi.nlm.nih.gov/gds) by search term “papillary renal cell carcinoma” (Searched on 10th of February 2016). After manually removing datasets in which PRCC1 and PRCC2 had not been separately classified, four datasets were obtained (Table 1). For the GSE26574 dataset, 22 PRCC1 and 12 PRCC2 samples were analyzed by expression profiling microarray using a GPL11433 platform without gender information [47]. In GSE7023 dataset, 14 PRCC1 and 16 PRCC2 samples were analyzed by expression profiling microarray using GPL4866 platform without gender information [48]. In GSE11024 and GSE2748 datasets, gender information was available for a total of 25 PRCC1 and 21 PRCC2 samples which were analyzed by expression profiling microarray using GPL6671 and GPL570 platforms, respectively [49–50]. In addition to microarrays datasets, one large PRCC dataset was available via TCGA-KIRP and consisted 77 PRCC1 and 86 PRCC2 with gender and clinical information [51]. This dataset had been analyzed by RNA-seq using Illumina Hiseq RNASeqV2-platform (http://cancergenome.nih.gov/).

### Data processing

Capacity computing environment (Finland CSC Taito-shell application server: https://research.csc.fi) was used for running integrative bioinformatics pipelines. For the four GEO datasets, the raw gene expression data and clinical information was downloaded from GEO by using GEOquery package in R (BioConductor; https://www.bioconductor.org/). GEOquery is an open-source and open-development software project [52, 16]. TCGA-assembler was applied for downloading and processing the TCGA-KIRP gene expression raw data and clinical information [53, 17]. After removing non-PRCC samples from the datasets, the remaining data was saved to a comma-delimited text-file by using Excel as described previously [54]. Objective quality control analysis was performed by importing the processed raw datasets in log-format into R by using MetaDE [54]. Largest interquartile range (IQR) method was used to calculate gene-wise expression. Non-expressed (30%) and non-informative (30%) genes were filtered out. MetaQC was applied to include or exclude the processed datasets as described previously [55, 18, 56].

### Data analysis

The MetaQC processed datasets were subjected to meta-analysis by combining *p*-values and effect sizes [54]. For the meta-analysis, the five datasets were combined and subjected to quality control and inclusion/exclusion criteria. These criteria consisted of internal quality control (IQC) index evaluating the homogeneity of coexpression and external quality control (EQC) index supervised by external pathway information, accuracy quality control indexes for genes (AQCg) and pathways (AQCp) and consistency of differential expression quality control (CQCg and CQCp) indexes that collectively depict the reproducibility and consistency of the data between the different individual the studies and results from the combined meta-analysis [57]. Three different meta-analysis methods for combining *p*-value; maxP, minP and roP were applied resulting in 1558, 1976 and 1758 differentially expressed (DE) genes, respectively (FDR = 0.05, Table 3) [20, 18]. For visualization, a heatmap plot of the DE genes (FDR = 0.05) was created by using MetaDE. A heatmap for pathway enrichment was provided by MetaPath packages, and MAPE_I was under *q*-value = 0.2 threshold [19]. Additionally, 2610 genes were obtained from a “REM” meta-analysis of the same raw data by combining effect sizes using MetaDE (FDR = 0.05) [58]. A Venn diagram was created to display an overlay of 1475 DE genes using Venny 2.1 (http://bioinfogp.cnb.csic.es/tools/venny/) [59]. The 1475 DE genes extracted from the overlay meta-analysis were further analyzed with DAVID 6.7 and PANTHER gene ontology to classify gene pathway, functions and interactions (https://david.ncifcrf.gov/) [60–61]. Protein-protein interactions of the identified DE genes were also analyzed using STRING v10 online tool that visualizes known and predicted protein-protein interactions (http://string.embl.de/) [62–63]. The gender comparison of PRCC2 samples were processed with MetaQC and MetaDE by combining the *p*-values of the raw data after PRCC1 cases were manually removed. PANTHER GO.Slim Molecular function analyses were performed as described earlier (http://pantherdb.org/) [64][65]. Expression analysis of *ITGA5*, *ITGA6* and *ITGB8* in Renal Cell Carcinomas and normal Kidney was obtained from Oncomine [26] and TCGA-KIRP.

## SUPPLEMENTARY MATERIALS FIGURES AND TABLES


