# Supplementary material for: Meta-analysis of gene expression and integrin-associated signaling pathways in papillary renal cell carcinoma subtypes
Source: Oncotarget. 2016 Oct 1;7(51):84178–89. doi: 10.18632/oncotarget.12390 (PMC5356653; doi:10.18632/oncotarget.12390)
Supplement: Supplementary file 1 [file oncotarget-07-84178-s001.pdf]

Meta-analysis of gene expression and integrin-associated signaling pathways in papillary renal cell carcinoma subtypes

Supplementary Materials

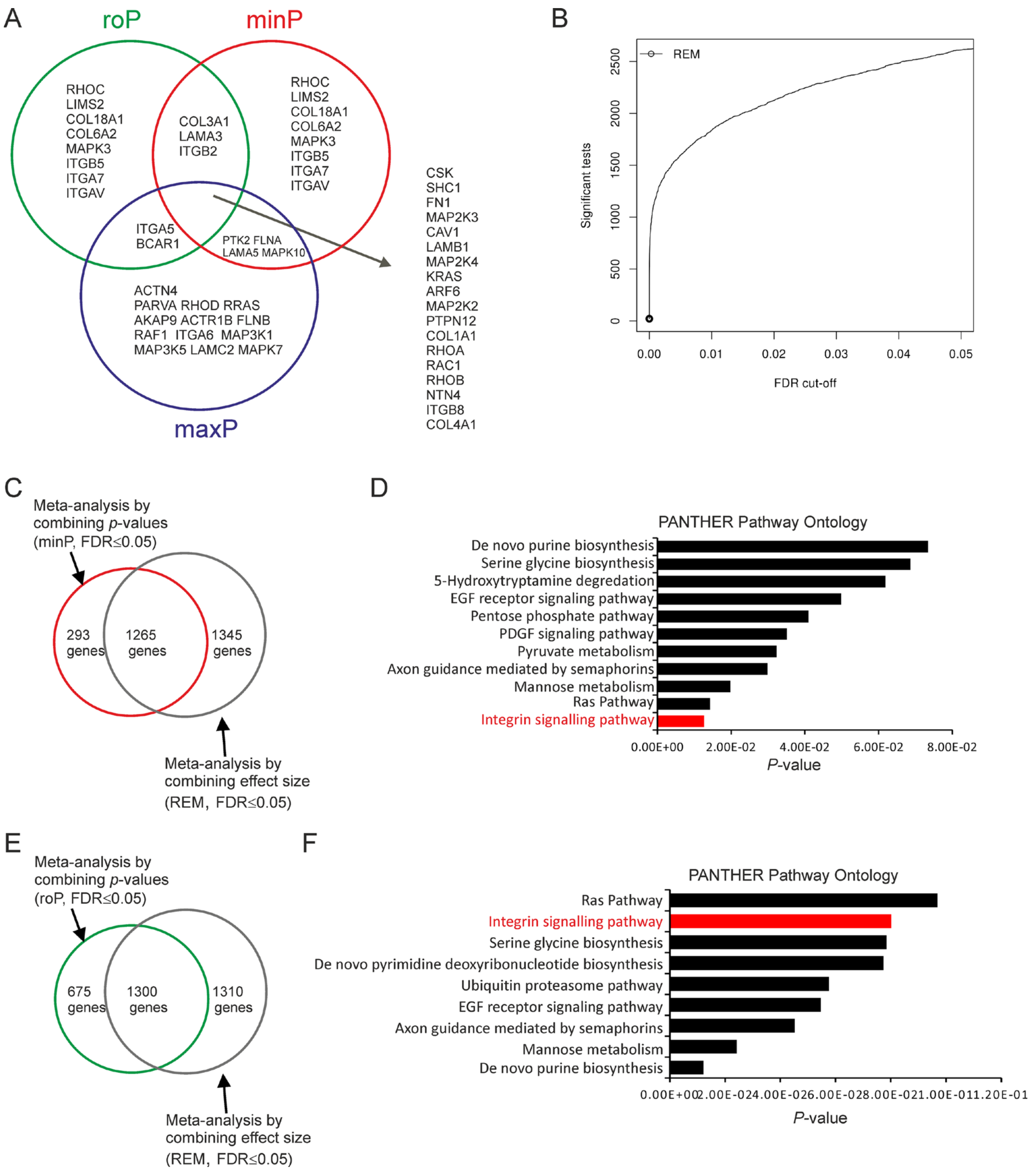

Supplementary Figure S1: Comparisons of the differentially expressed genes between PRCC1 and PRCC2 obtained by using different meta-analysis methods. (**A**) A Venn Diagram shows the overlay of DE integrin pathway genes detected by the three meta-analyses using roP, minP or maxP criteria for the *p*-values. (**B**) 2610 differentially expressed genes were plotted as a function of false discovery rate FDR in the “REM” meta-analysis that combines the effect sizes. (**C**) A Venn Diagram showing the overlap of DE genes detected by the two meta-analyses, one combining the *p*-values (minP) and another combining the effect sizes (REM). (**D**) PANTHER pathway ontology analysis highlighting integrin pathway as the most enriched pathway in combined meta-analysis (minP/REM). (**E**) A Venn Diagram showing the overlap of DE genes detected by the two meta-analyses, one combining the *p*-values (roP) and another combining the effect sizes (REM). (**F**) PANTHER pathway ontology analysis of the overlapping DE genes from E. highlighting integrin pathway in red.

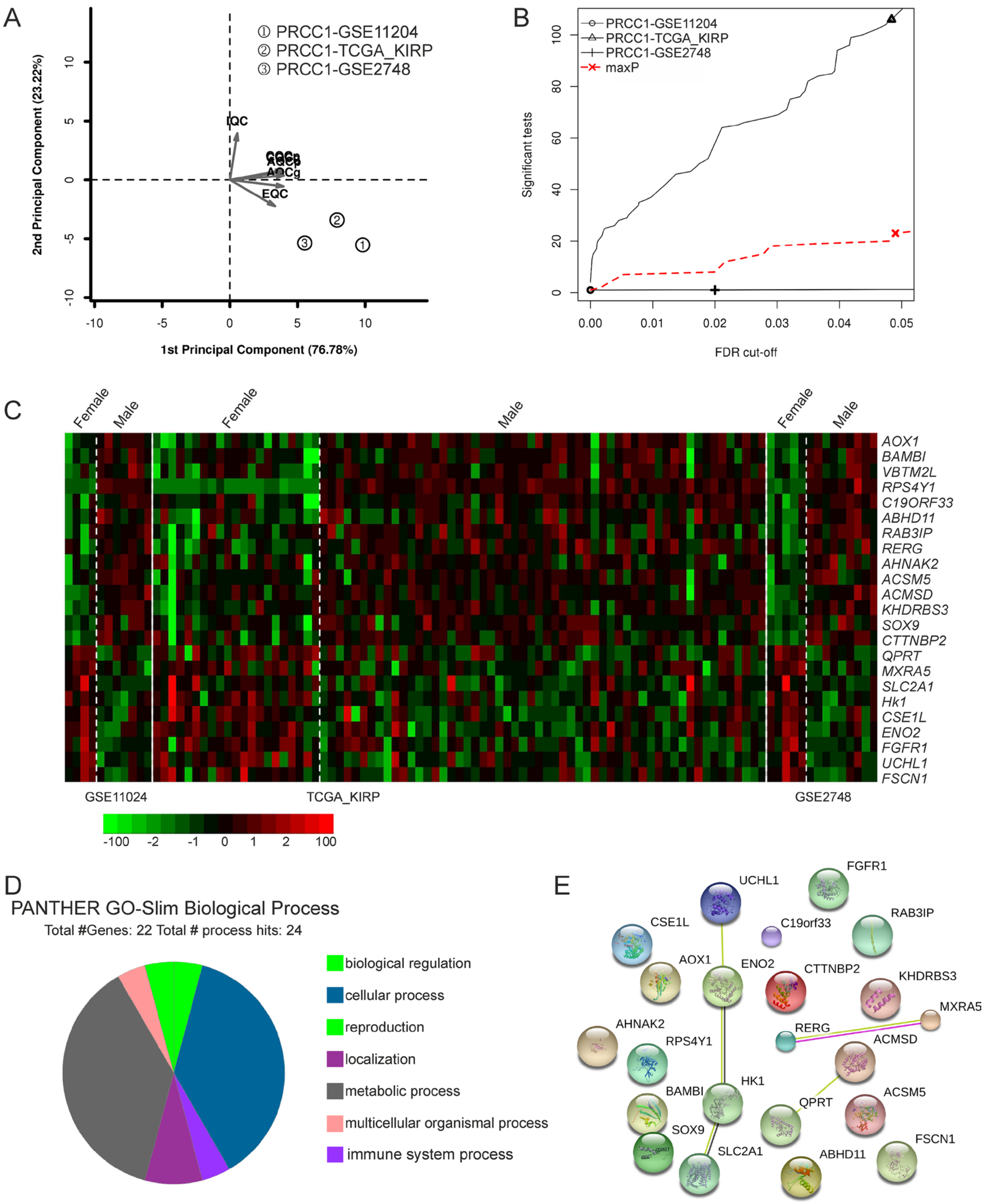

Supplementary Figure S2: Comparative meta-analysis of DE genes in female and male PRCC1 patients. (**A**) PCA biplot of quality control measures in the three PRCC studies after removing non-PRCC1 samples. (**B**) The detection competency curves of DE genes plotted against false-discovery rate in analyses of the three individual datasets and the maxP meta-analyses approaches. (**C**) A heat map representation of DE genes (FDR = 0.05) between female and male PRCC1 patients. (**D**) Molecular function analysis of the 23 DE genes revealed by the meta-analysis. (**E**) Protein-protein interaction network analysis of the 23 DE genes in female and male PRCC1 patients.
